# Relationship between the Levels of lncRNA H19 in Plasma and Different Adipose Tissue Depots with Patients’ Response to Bariatric Surgery

**DOI:** 10.3390/life12050633

**Published:** 2022-04-25

**Authors:** Marina S. Artemyeva, Ludmila B. Vasileva, Yi Ma, Kirill A. Kondratov, Anton V. Fedorov, Anna A. Kostareva, Sofia E. Lapshina, Anna D. Anopova, Nikolai P. Likhonosov, Alexander E. Neymark, Alina Yu. Babenko, Evgeny V. Shlyakhto

**Affiliations:** Federal State Budgetary Institution “V.A. Almazov National Medical Research Centre” of the Ministry of Health of the Russian Federation, 197341 Saint Petersburg, Russia; marinaart888@mail.ru (M.S.A.); 79213735067@yandex.ru (L.B.V.); mayi29082@yandex.ru (Y.M.); kondratovk.kirill@yandex.ru (K.A.K.); anna.kostareva@ki.se (A.A.K.); s.e.lapshina@gmail.com (S.E.L.); anchylove@mail.ru (A.D.A.); nick.pino@mail.ru (N.P.L.); neymark_ae@almazovcentre.ru (A.E.N.); alina_babenko@mail.ru (A.Y.B.); e.shlyakhto@almazovcentre.ru (E.V.S.)

**Keywords:** obesity, impaired carbohydrate metabolism, bariatric surgery, biomarkers, lncRNA H19

## Abstract

Bariatric surgery represents a widespread approach to treating morbid obesity. The search for biomarkers to identify patients to whom this type of treatment will be most effective is needed. Our aim was to characterize the relationship of levels of lncRNA H19 in plasma and different adipose tissue depots with patients’ response to bariatric surgery. The study includes control subjects, patients with obesity and patients with obesity accompanied by impaired carbohydrate metabolism (ICM). Quantitative analysis of lncRNA H19 levels has been performed using qPCR in plasma and subcutaneous (SAT) and visceral adipose tissue (VAT). Patients with obesity without ICM have higher levels of lncRNA H19 in VAT compared to SAT, and higher levels of lncRNA H19 in SAT compared to SAT of control individuals. One year after the intervention, levels of lncRNA H19 decreased in SAT of patients with obesity without ICM. The preoperative level of lncRNA H19 in VAT demonstrates a positive correlation with excess weight loss and a negative correlation with initial BMI. In conclusion, ICM affects expression of lncRNA H19 in SAT of patients with obesity. The preoperative level of lncRNA H19 in VAT can be used to predict excess weight loss in patients with obesity after bariatric surgery.

## 1. Introduction

Obesity is one of the most common diseases worldwide [1] and a leading risk factor for many chronic disorders such as type 2 diabetes mellitus, cardiovascular and renal diseases, non-alcoholic fatty liver disease, and many cancers [2]. The prevalence of obesity has tripled in just the last half century [3]. The accumulated data indicate that various factors contribute to the development of obesity and its complications: social, phenotypic, psychological, and genetic. Nutrition and certain characteristics of therapy play an important role in the regulation of molecular pathways involved in the control of metabolic processes.

Currently, the investigation of molecular pathways associated with the development of obesity is aimed at understanding the mechanisms of underlying metabolic changes as well as searching for new methods of treatment and predicting its effectiveness. The most effective treatment for morbid obesity is bariatric surgery. This intervention achieves a substantial reduction in body weight and has a positive effect on obesity-related pathologies [4,5]. However, bariatric interventions are not equally effective for all patients, and both the degree and the rate of weight loss can vary considerably [6]. Early prediction of response to treatment allows the selection of patients for whom this type of intervention will be the most effective and ensures the personalization of treatment of patients with obesity.

One of the promising classes of biomarkers of the therapy response are non-coding RNAs, including microRNAs and long non-coding RNAs (lncRNAs), since these biomolecules are present and stable in physiological fluids, and changes in their levels can be quantitatively measured using a relatively cheap and fast real-time PCR technique. LncRNAs are transcripts longer than 200 nucleotides without protein-coding function [7,8,9]. Recent studies have shown the participation of lncRNAs in the regulation of the endocrine system functions, including the metabolism of adipose tissue [10,11]. One of the representatives of the class of lncRNAs is long non-coding RNA H19 (lncRNA H19), initially attracting attention as a cell growth regulator with maximum expression in various tissues during embryogenesis [9]. Further studies have demonstrated its role in the regulation of adipose tissue metabolism as a selective modulator of functions of brown adipose tissue. In an experimental model of obesity in mice, it was shown that the levels of lncRNA H19 change in the brown adipose tissue during development of obesity, and that increased expression of lncRNA H19 protects animals from the development of high-fat diet-induced obesity [12]. In humans, lncRNA H19 expression is detected in both subcutaneous adipose tissue (SAT) and visceral adipose tissue (VAT), and its level positively correlates with markers of adipose tissue browning such as mRNA of UCP1; in people with obesity, SAT and VAT levels of lncRNA H19 decrease with increasing BMI [12]. Furthermore, lncRNA H19 was found in plasma and its levels in circulation correlate with various pathological conditions [13]. lncRNA H19 is associated with insulin sensitivity, mitochondrial bioenergetics [13], provides postpartum expansion of pancreatic beta-cell mass [14], and counteracts the development of left ventricular hypertrophy [15].

In the present study, lncRNA H19 is selected as a candidate biomarker to predict patient response to bariatric interventions. Since adipose tissue from different depots makes different contributions to the pathogenesis of obesity and the characteristics of the response to therapy [16], it is of interest to assess the levels of lncRNA H19 in SAT and VAT depots as well as in plasma.

The aims of the present study were to characterize the relationship of levels of lncRNA H19 in plasma and different adipose tissue depots with response to bariatric surgery in patients with obesity.

## 2. Materials and Methods

### 2.1. Subjects

*Inclusion criteria:* Patients with a BMI of more than 40 kg/m^2^ or with a BMI of 35–40 kg/m^2^ in the presence of concomitant diseases, the improvement of which should be expected with a decrease in body weight (diabetes mellitus, diseases of the cardiovascular system, joint damage, associated psychological problems). Gender—female; age—25–65; informed consent of the patient to participate in this study; informed consent to perform a biopsy of the adipose tissue.

*Non-inclusion criteria:* Presence of type 1 diabetes mellitus. Acute coronary syndrome; acute cerebrovascular accident in the last 2 months. Decompensation of chronic heart failure. Chronic kidney disease requiring hemodialysis therapy and chronic kidney disease at the stage of proteinuria. History of malignant neoplasms. The presence of ulcers of the lower extremities as part of the diabetic foot syndrome. History of bariatric surgery or surgery on the organs of the gastrointestinal tract, leading to malabsorption. The presence of liver diseases with an increase in the level of alanine aminotransferase, aspartate aminotransferase, more than 3 times higher than the upper limit of the norm. Systemic glucocorticosteroid therapy or change in thyroid hormone dosage in the last 6 weeks.

*Exclusion criteria:* Loss of contact with the patient and, consequently, the impossibility of collecting information on weight dynamics.

The group of patients for this study was formed using a database of patients with a high degree of obesity undergoing treatment of bariatric surgery at the Federal State Budgetary Institution “V.A. Almazov National Medical Research Centre” of the Ministry of Health of the Russian Federation, St. Petersburg. The study protocol was approved by the Ethics Committee of the V.A. Almazov National Medical Research Centre of the Ministry of Health of Russia (protocol No. 63 dated 14 April 2014).

The study includes 3 groups of individuals. Group C—control subjects (n = 10); group OB—patients with obesity (n = 19); group OBICM—patients with obesity accompanied by impaired carbohydrate metabolism (n = 11).

### 2.2. Collection of Adipose Tissue and Plasma Samples

In group C, samples of plasma and SAT were collected. In OB and OBICM groups, samples of plasma, SAT, and VAT were collected.

In groups OB and OBICM, samples of blood for plasma isolation were collected no more than 1 day before surgery and 1 year after the intervention; VAT samples were collected during bariatric surgery; and SAT samples were collected during bariatric surgery and 1 year after the intervention.

During bariatric surgery, SAT samples were collected from the peri-umbilical region by an incisional method through a 1.5 cm skin incision, and a section of adipose tissue with a volume of 1.5–2 cm^3^ was isolated using scissors; VAT samples were collected using an ultrasonic scalpel by resection of the marginal fragment of the greater omentum with a volume of 3–4 cm^3^. In group C, as well as in groups OB and OBICM, 1 year after the intervention, samples of SAT were collected by the aspiration method, using a syringe for injection.

Blood plasma was obtained according to the previously described method [17]. Collected adipose tissue samples were washed once in saline before freezing. Plasma and adipose tissue samples were frozen in liquid nitrogen no later than 30 min after preparation and then stored at −80 °C.

### 2.3. Anthropometric Parameters

When included in the study, all patients underwent a physical examination: measurement of height in cm, body weight in kg, at no more than 1 day before the operation. The follow-up examinations to determine the current body weight of patients were performed 1 year after the bariatric surgery.

Body mass index (BMI) and percent of excess weight loss (%EWL) were calculated according to formulas:

BMI = body weight/(height)^2^, [kg/m^2^]

%EWL = 100% × (initial body weight − current body weight)/(initial body weight − 25 × (height)^2^), [%]

### 2.4. RNA Isolation and Quantitative Real-Time PCR

Total RNA was isolated from adipose tissue samples using the ExtractRNA reagent (Evrogen, Russia) according to the manufacturer’s recommendations. RNA precipitation was carried out in the presence of 8 μg of the co-precipitant GlycoBlue (Thermo Fisher Scientific, Waltham, MA, USA). Total RNA from 100 μL of plasma was isolated using the Trizol LS reagent (Thermo Fisher Scientific, Waltham, MA, USA), according to the previously described method [18]. RNA precipitates from adipose tissue and from plasma were dissolved, respectively, in 20 and 30 μL of deionized water treated with diethyl pyrocarbonate. Aqueous solutions of RNA were stored at −80 °C. The RNA concentration was determined using a ND-1000 Nanodrop spectrophotometer (Thermo Fisher Scientific, Waltham, MA, USA)

Analysis of the lncRNA H19 relative levels was performed using quantitative real-time PCR. Reverse transcription with random primers was performed in the Veriti 96-Well Thermal Cycler model 9902 (Thermo Fisher Scientific, Waltham, MA, USA) using the MMLV RT kit (Evrogen, Moscow, Russia) according to the manufacturer’s recommendations. Reverse transcription reactions used 4 ng of RNA from adipose tissue or 8 μL of an aqueous solution of RNA from plasma. Amplification and registration of the fluorescent signal was performed in a 7500 Real-Time PCR System (Thermo Fisher Scientific, Waltham, MA, USA) using the qPCRmix-HS SYBR+LowROX reagent (Evrogen, Russia) according to the manufacturer’s recommendations. To detect lncRNA H19, the following primers were used: H19_F 5’-ATGACATGGTCCGGTGTGAC-3’, H19_R 5’-GAAACAGACCCGCTTCTTGC-3’. The following primers were used to detect the reference GAPDH transcript: GAPDH_F 5’-AATGAAGGGGTCATTGATGG-3’, GAPDH_R 5’-AAGGTGAAGGTCGGAGTCAA-3’.

The absence of inhibition of enzymatic reactions was monitored by detection of a reference GAPDH transcript in adipose tissue samples and spiked-in cel-miR-39 RNA sequence in plasma samples. Spiked-in cel-miR-39 RNA sequence was detected as described earlier [19].

Since plasma circulating RNA was isolated from samples of the same volume, no additional normalization of the values of lncRNA H19 quantification cycles (Cq) from plasma was performed. In the case of adipose tissue samples, the values of the lncRNA H19 quantification cycles were normalized as follows. Cq = Cq__H19_ − (Cq__GAPDH_ − Cq__GAPDH_median_), where Cq__H19_ is the value of the lncRNA H19 quantification cycle in a particular sample, Cq__GAPDH_ is the value of the GAPDH quantification cycle in the same sample, and Cq__GAPDH_median_ is the median of Cq _GAPDH_ of all adipose tissue samples. The relative amounts of lncRNA H19 were determined as 2^(Cq_max−Cq)^, where Cq is the normalized value of the lncRNA H19 quantification cycle in a particular sample and Cq_max is the maximum of the normalized values of the lncRNA H19 quantification cycle in all samples.

### 2.5. Statistical Analysis

Statistical analysis and visualization of the results were performed using the GraphPad Prism 5 software. Quantitative data are presented in the format of the median and interquartile range (25%; 75%). To analyze the differences in parameter values, the nonparametric Wilcoxon and Mann–Whitney tests were used. To study the relationship between the parameters, the Spearman correlation coefficient and its actual level of significance (*p*-value) were calculated. A *p*-value < 0.05 was considered statistically significant.

## 3. Results

### 3.1. Expression of lncRNA H19 in Adipose Tissue of Patients with Obesity Is Associated with the Presence of ICM

**Figure 1:**
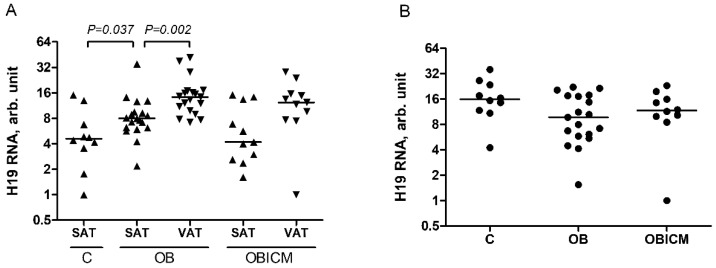
Relative levels of lncRNA H19 in SAT and VAT samples (**A**) and plasma (**B**). C—control subjects, OB—patients with obesity, OBICM—patients with obesity accompanied by impaired carbohydrate metabolism. Mann—Whitney test was used to calculate p-values. Logarithmic scale is used for the Y axis.

LncRNA H19 was detected in plasma and adipose tissue of patients with obesity. Quantitative analysis showed that in contrast to patients from OBICM group, patients from OB group have 1.5 times higher levels of lncRNA H19 in VAT compared to SAT (*p* = 0.002) and 1.6 times higher levels of lncRNA H19 in SAT compared to SAT of control individuals (*p* = 0.037) (Figure 1A). Levels of lncRNA H19 in plasma do not differ between C, OB, and OBICM groups (Figure 1B).

**Figure 2:**
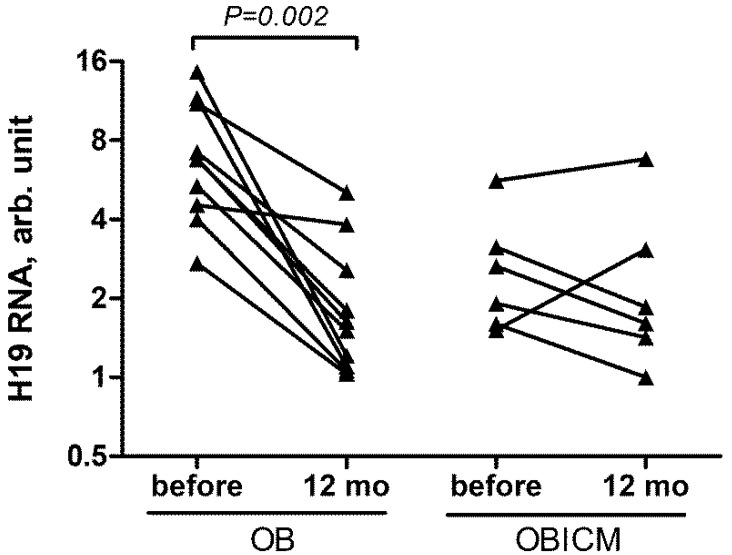
Relative levels of lncRNA H19 in SAT samples before and 1 year after bariatric surgery. OB—patients with obesity, OBICM—patients with obesity accompanied by impaired carbohydrate metabolism. Wilcoxon test was used to calculate *p*-values. Logarithmic scale is used for the Y axis.

One year after the intervention, levels of lncRNA H19 in SAT from OB group decrease 3.7 times (*p* = 0.002), levels of lncRNA H19 in SAT from OBICM group remain unchanged (Figure 2), and levels of lncRNA H19 in plasma from OB and OBICM groups do not change (data not shown).

### 3.2. Preoperative Level of lncRNA H19 in VAT Correlates with %EWL and Initial BMI

Demographic and clinical characteristics of participants as well as anthropometric characteristics before and 1 year after the bariatric surgery are presented in Table 1.

## 4. Discussion

One year after bariatric intervention, all studied patients with obesity experienced substantial weight loss. At the same time, we found that %EWL, an anthropometric parameter used to evaluate the therapy effectiveness, reaches values comparable to those described in previous studies and demonstrate known association with initial BMI [18,20,21].

In this work, lncRNA H19 was detected in SAT and VAT, as well as in the plasma of the patients with obesity. Differences in the levels of lncRNA H19 from VAT and SAT, as well as the lack of correlation between them, is an indicator of different organization of molecular pathways in adipose tissue from these two localizations [22,23].

Differences in the expression of lncRNA H19 in SAT were revealed between patients with obesity and obesity accompanied by ICM. Namely, in contrast to patients with obesity accompanied by ICM, patients with obesity have higher levels of SAT lncRNA H19, which decreased in response to bariatric intervention. This indicates that ICM has some effect on the expression of lncRNA H19 in SAT of patients with obesity. One of the factors affecting the differences in the expression of lncRNA H19 in patients with obesity and obesity accompanied by ICM may be a decrease in energy deficit at higher glycemic levels.

LncRNA H19 can enter the circulation system either as a result of active secretion, or as a result of passive release from the dead cells. The lack of correlation between the levels of lncRNA H19 in plasma and adipose tissue makes it impossible to predict the levels of lncRNA H19 in the adipose tissue based on its levels in circulation. It also suggests the existence of additional cellular sources of the circulating form of lncRNA H19. One of these could be skeletal muscles, which exhibit a high level of lncRNA H19 [24].

The negative correlation between levels of lncRNA H19 in VAT and initial BMI highlights the role of VAT in the development of obesity and supports the hypothesis of the participation of lncRNA H19 in the molecular pathways of obesity development [12]. At the same time, the revealed correlation between the lncRNA H19 levels in VAT and %EWL is most likely a consequence of the dependence of lncRNA H19 levels and this anthropometric parameter on the initial BMI.

The present study has some limitations.

1. Due to small number of enrolled patients this should be considered a pilot study.
2. The absence of VAT samples in the control group made it impossible to compare VAT lncRNA H19 levels in control subjects and patients with obesity.
3. Patients studied had a fairly wide range of initial BMI. It is impossible to exclude the influence of initial BMI on the parameters studied, as well as the response of the patients to bariatric interventions. To exclude the effect of these factors, subgroups of patients with an initial BMI in a narrow range of selected values can be used in the future.
4. Expression of lncRNA H19 can also be affected by lifestyle factors which were not considered in this study.

## 5. Conclusions

ICM affects expression of lncRNA H19 in SAT of patients with obesity. The preoperative level of lncRNA H19 in VAT correlates with %EWL one year after surgery; therefore, this parameter can be used as a biomarker to predict patient response to bariatric treatment of obesity.

## Figures and Tables

**Table 1:** Demographic, clinical, and anthropometric characteristics of participants.

Parameter\Group	C (Control Subjects)	OB (Patients with Obesity)	OBICM (Patients with Obesity Accompanied by Impaired Carbohydrate Metabolism)
Age, years	35.5 [29.0; 44.3]	42.0 [35.0; 43.0]	44.0 [36.0; 52.0]
Gender	F; n = 10; 100%	F; n = 19; 100%	F; n = 11; 100%
Impaired glucose tolerance	NA	NA	n = 3; 27.3%
Type 2 diabetes mellitus	NA	NA	n = 8; 72.7%
Body weight before surgery, kg	59.5 [54.3; 65.0]	124.0 [100.0; 148.0]	126.0 [115.0; 141.0]
Body weight 1 year after surgery, kg	NA	81.4 [72.3; 104.3]	88.0 [75.5; 94.5]
BMI before surgery, kg/m^2^	22.0 [21.0; 23.6]	41.5 [37.2; 52.2]	44.8 [39.3; 57.4]
BMI 1 year after surgery, kg/m^2^	NA	29.2 [26.3; 38.5]	32.8 [26.1; 37.9]
%EWL 1 year after surgery	NA	58.1 [47.9; 74.8]	50.6 [45.6; 74.6]

Correlation analysis was used to assess the relationships between initial BMI, %EWL 1 year after surgery and lncRNA H19 levels in different tissue. No statistically significant correlations were found between lncRNA H19 levels in plasma, VAT, and SAT (data not shown). Preoperative level of lncRNA H19 in VAT demonstrates a positive correlation with %EWL (r = 0.59, *p* = 0.013) and a negative correlation with initial BMI (r = −0.62, *p* = 0.008). LncRNA H19 levels in SAT and plasma do not correlate with either %EWL or initial BMI. A negative correlation was found between initial BMI and %EWL (r = −0.68, *p* = 0.003) (Table 2).

**Table 2:** Relationship of lncRNA H19 levels from different tissues with initial BMI and %EWL.

Anthropometric Parameter	Patients Subgroups	Initial BMI		H19 from VAT		H19 from SAT		H19 from Plasma	
		r	*p*	r	*p*	r	*p*	r	*p*
Initial BMI	OB + OBICM (n = 30)	NA	NA	**−0.62**	**0.008**	−0.18	0.485	0.03	0.905
	OB (n = 19)	NA	NA	**−0.58**	**0.048**	0.06	0.846	0.12	0.713
%EWL 1 year after surgery	OB + OBICM (n = 30)	**−0.68**	**0.003**	**0.59**	**0.013**	−0.09	0.707	−0.29	0.273
	OB (n = 19)	**−0.68**	**0.015**	0.45	0.145	−0.25	0.430	−0.45	0.145

## Data Availability

All data generated or analyzed during this study are included in this article and its supplementary material files. Further enquiries can be directed to the corresponding author.
